# MDR Bacteremia in the Critically Ill During COVID-19: The MARTINI Study

**DOI:** 10.3390/pathogens14111152

**Published:** 2025-11-12

**Authors:** Karolina Akinosoglou, Christina Petropoulou, Vasiliki Karioti, Sotiria Kefala, Dimitrios Bousis, Vasiliki Stamouli, Fevronia Kolonitsiou, George Dimopoulos, Charalambos Gogos, Foteini Fligou

**Affiliations:** 1 Department of Medicine, University of Patras, 26504 Patras, Greece; chripetr96@gmail.com (C.P.); sotiria_kefala@yahoo.gr (S.K.); kolonits@upatras.gr (F.K.); cgogos@med.upatras.gr (C.G.); fflig@upatras.gr (F.F.); 2 Department of Internal Medicine, University General Hospital of Patras, 26504 Patras, Greece; 3 Division of Infectious Diseases, University General Hospital of Patras, 26504 Patras, Greece; 4 Department of Tourism Management, University of Patras, 26504 Patras, Greece; vaskar@upatras.gr; 5 Intensive Care Unit, University General Hospital of Patras, 26504 Patras, Greece; dimitrisbousis@gmail.com; 6 Department of Microbiology, University General Hospital of Patras, 26504 Patras, Greece; vstam@pgnp.gr; 7 3rd Department of Critical Care, Evgenidio Hospital, Medical School, National and Kapodistrian University of Athens, 11528 Athens, Greece; gdimop@med.uoa.gr

**Keywords:** multidrug resistant pathogens, antimicrobial resistance, COVID-ICU, critically ill, non-COVID ICU, SARS-CoV-2

## Abstract

Multidrug-resistant (MDR) bloodstream infections (BSIs) constitute a major challenge in intensive care units, with the COVID-19 pandemic compromising infection control and potentially increasing MDR incidence. Comparative data between COVID and non-COVID ICU populations remain limited. The MARTINI study is a retrospective observational analysis held in a tertiary hospital during the COVID-19 pandemic (2020–2022) encompassing adult patients with MDR BSIs admitted to COVID and non-COVID ICUs. Demographics, comorbidities, severity scores, microbiology, resistance patterns, and outcomes were accessed and compared. A binary logistic regression model and multivariate regression was performed to identify independent predictors of ICU mortality. Among the study’s 156 patients (106 COVID-ICU, 50 non-COVID-ICU), COVID-ICU patients were significantly older with higher comorbidity and severity scores. Gram-negative pathogens predominated in both cohorts, mainly *Acinetobacter baumannii* and *Klebsiella pneumoniae*, with comparable resistance mechanisms. Timing of bacteremia onset and initiation of appropriate therapy did not differ between groups. However, ICU mortality was markedly higher in COVID-ICU patients (74.5% vs. 38%, *p* < 0.001). Age, SOFA score, the presence of systemic inflammation (SIRS) and COVID-19 infection were identified as independent predictors of mortality. Although pathogen distribution and resistance were similar across groups, COVID-ICU patients experienced significantly poorer outcomes. Strengthened infection control and timely and targeted antimicrobial therapy are essential to diminish MDR bacteremia risk in critically ill populations.

## 1. Introduction

Antimicrobial resistance (AMR) has emerged as a public health threat, with significant implications for patient outcomes, healthcare costs, and global health safety. According to recent estimates, AMR is associated with nearly five million deaths annually, with at least 1.27 million directly attributed to drug-resistant infections in 2019 alone [1]. The burden is especially profound in intensive care units (ICUs), where critically ill patients are more vulnerable to nosocomial infections due to frequent invasive procedures, prolonged hospital stays, and immunosuppressive therapies. In this context, bloodstream infections (BSIs) caused by multidrug-resistant (MDR) organisms are most frequently encountered [2]. These infections are not only associated with increased morbidity and mortality but also with limited therapeutic options and prolonged hospitalization.

The COVID-19 pandemic, caused by the novel coronavirus SARS-CoV-2, led to an unprecedented strain on healthcare systems worldwide. During the early stages of the pandemic, ICUs were overwhelmed by critically ill patients with severe respiratory failure requiring mechanical ventilation. This scenario contributed to significant disruptions in routine infection control practices and antimicrobial stewardship programs [3]. Multiple factors during the pandemic are believed to have accelerated the emergence and transmission of MDR pathogens in ICUs. These include excessive and often empirical use of broad-spectrum antibiotics, shortages of personal protective equipment (PPE), overburdened healthcare workers, and the frequent use of immunomodulatory agents like corticosteroids and tocilizumab [4,5]. Disruptions in routine microbiological surveillance, delays in diagnostic stewardship, and the prioritization of COVID-19 care over infection prevention activities may have also played critical roles. Notably, several studies have reported a surge in healthcare-associated infections (HAIs) during COVID-19 surges, many of which were caused by MDR pathogens [6].

A growing body of evidence suggests that MDR BSIs increased substantially during the COVID-19 pandemic, particularly among patients admitted to COVID-designated ICUs [7]. A multicenter Italian study observed a significant increase in carbapenem-resistant *Klebsiella pneumoniae* colonization during the pandemic period compared to the pre-pandemic baseline [8]. Similarly, a Greek ICU study reported a higher incidence of bloodstream infections due to *Acinetobacter baumannii* and *Pseudomonas aeruginosa*, both of which exhibit extensive drug resistance in patients with COVID-19 [9]. The frequent use of central venous catheters, prolonged mechanical ventilation, and immunosuppression in these patients may have contributed to these trends. Furthermore, the immunopatholo-gical features of severe COVID-19—including cytokine storm and endothelial dysfunction—might increase susceptibility to secondary bacterial infections [10].

The pandemic highlighted the vulnerabilities in infection control infrastructure and antimicrobial stewardship programs. With infection prevention staff redeployed to manage COVID-19 protocols, routine surveillance, hand hygiene audits, and environmental cleaning practices were deprioritized. These lapses, combined with increased antibiotic prescribing even in the absence of confirmed bacterial co-infections, contributed to AMR amplification. Furthermore, initial uncertainty about bacterial co-infection rates in COVID-19 led many clinicians to initiate empirical antibiotic treatment early in hospitalization, despite subsequent evidence indicating a relatively low incidence of confirmed bacterial co-infections at presentation [11].

However, despite the known rise in MDR BSIs during the pandemic, direct comparisons between COVID and non-COVID ICU populations remain limited [12]. Despite the increasing number of reports describing MDR bacteremias during the COVID-19 pandemic, there remains a lack of robust, comparative studies evaluating their incidence and impact in COVID versus non-COVID ICU populations. The few studies that exist are often limited by small sample sizes, retrospective designs, or lack of standardized MDR definitions. To address this gap, the present study aims to systematically compare the epidemiology, antimicrobial resistance profiles, clinical outcomes, and potential differential risk factors for MDR bloodstream infections in patients admitted to COVID and non-COVID ICUs between 2020 and 2022.

## 2. Materials and Methods

This is a retrospective observational study that analyzed data from patients hospitalized in the COVID and non-COVID ICUs of a tertiary university hospital from April 2020 to December 2022. Our choice of a retrospective observational design was primarily due to the need for evaluation of clinical data collected during the COVID-19 pandemic, when prospective enrollment was not feasible, due to the pandemic’s duration and the conduction of the study after its end. This design allowed us to analyze a large number of patients admitted during a defined period and identify potential prognostic factors based on real-world data.

The study was conducted in compliance with principles of declaration of Helsinki for good clinical research practice and the study protocol received approval from the local Research Ethics Committee and Institutional Review Board (325/13.07.2023). In line with the hospital’s Research Ethics Committee policy during the pandemic, informed consent requirements for observational studies were waived to help reduce the risk of disease transmission, given that GDPR was ensured. Nonetheless, before including a patient in the study, a phone call was made to the patient’s legal representative to request permission for data collection.

### 2.1. Study Population

Eligible patients included adults hospitalized from April 2020 to December 2022, thus coinciding with the major COVID-19 pandemic waves in the COVID and non-COVID ICUs. Adult patients (aged 18 years or older) experiencing their first episode of hospital-acquired bloodstream infection (HABSI) with MDR bacterial or fungal pathogen and receiving treatment in the ICU were included in the study. HABSI was identified by a positive blood culture obtained at least 48 h after hospital admission. The study population comprised both patients whose blood cultures were drawn while in the ICU (indicating ICU-acquired HABSI) and those who were transferred to the ICU within 48 h for HABSI management.

The classifications MDR (multidrug-resistant), XDR (extensively drug-resistant), and PDR (pandrug-resistant) were applied according to established definitions. MDR refers to acquired resistance to at least one drug in three or more antimicrobial classes, which include aminoglycosides, antipseudomonal carbapenems, antipseudomonal fluoroqui-nolones, antipseudomonal penicillins combined with beta-lactamase inhibitors, extended-spectrum cephalosporins, folate pathway inhibitors, penicillins with beta-lactamase inhibitors, polymyxins, and tetracyclines. XDR describes bacteria that are resistant to at least one agent in all but two or fewer antimicrobial categories, meaning they remain susceptible to only one or two classes. PDR is defined as resistance to all tested agents across all antimicrobial categories [2]. Isolates were classified as susceptible when they did not meet the criteria for multidrug-resistant (MDR), extensively drug-resistant (XDR), or pandrug-resistant (PDR) categories, according to the definitions proposed by Magiorakos et al. [2]. In essence, susceptible isolates were those showing resistance to fewer than three antimicrobial categories, and those were excluded from our study.

Blood cultures yielding common skin flora (e.g., coagulase-negative staphylococci, *Corynebacterium* spp., *Bacillus* spp., etc.) were included only if two or more cultures showed identical antimicrobial susceptibility patterns or if there was compelling clinical evidence suggesting a true infection source (e.g., confirmation of infected material linked to the HABSI). Each HABSI case involving typical skin contaminants was thoroughly evaluated by an infectious diseases specialist. For this analysis, only the first bloodstream infection occurring within the eligibility timeframe was considered. Cases were excluded if they involved community-acquired bloodstream infections, had skin contaminants that did not meet inclusion criteria, exceeded 3 months of ICU stay or were missing essential outcome data (such as dates of infection onset, hospital/ICU admission, discharge or death, pathogen identification, treatment details including antimicrobial therapy and source control).

### 2.2. Data Collection

Data for each eligible patient was extracted from the institution’s electronic medical records, microbiology laboratory systems, and patient files. Demographic and baseline clinical characteristics included variables such as age, sex, somatometric measurements, comorbidities, cause of admission (infection, trauma, COVID pneumonia, etc.), duration of ICU stay, intubation, clinical outcome, laboratory findings on the day of first positive blood culture, severity scores (CCI, SOFA, PITT, APACHE II), and serious complications. The microbiological profile included the first MDR-positive blood culture, as well as, reference to following MDR isolates or polymicrobial cultures, and the intervals between positive blood culture, initiation of appropriate antibiotic(s), and the first negative culture or the patient’ death. In cases where the same pathogen was isolated more than once from the same patient, only the first isolate was included in the analysis. Duplicate isolates with differing resistance profiles were identified in the course of long hospitalizations, but again only the first isolate was registered, being aware of the possibility of excluding more complex resistance profiles.

### 2.3. Patient Management

All patients hospitalized with COVID infection were treated for SARS-CoV-2 according to international and national guidelines at the time of their admission [13]. In COVID-19 and non-COVID-19 patients, empirical broad-spectrum antibiotic therapy was initiated upon suspicion of sepsis either before or upon ICU admission, in line with Surviving Sepsis Campaign International Guidelines and in agreement with daily infectious diseases consultations in critically ill patients [14]. Upon pathogen identification, antimicrobial de-escalation was performed.

### 2.4. Pathogen Identification

Pathogens were identified using the VITEK^®^ 2 system with Gram-positive, Gram-negative, and fungi identification cards (bioMérieux, Marcy-l’Étoile, France). The antimicrobial susceptibility testing included agents such as amikacin, gentamicin, tobramycin, ciprofloxacin, levofloxacin, imipenem, meropenem, tigecycline, trimethoprim/sulfamethoxazole, and colistin. Between 2020 and 2022, minimum inhibitory concentrations (MICs) were also assessed for ceftazidime, cefepime, ampicillin/sulbactam, piperacillin/tazobactam, and minocycline. Similarly, voriconazole, amphotericine B, antidulafungin, caspofungin, and micafungin were assessed for fungal pathogens. The selection of antimicrobial agents was guided by recommendations from the European Committee on Antimicrobial Susceptibility Testing (EUCAST), the European Center for Disease Prevention and Control (ECDC), and the Centers for Disease Control and Prevention (CDC). According to EUCAST criteria, isolates were classified as either susceptible (including both “susceptible” and “susceptible, increased exposure”) or resistant. MIC determination for colistin was performed using the broth microdilution method (SensiTest™ Colistin, Liofilchem, Roseto degli Abruzzi, Italy), in accordance with recommended practices.

### 2.5. Statistical Analysis

Statistical analysis was performed using IBM SPSS Statistics version 28. Categorical variables were expressed as absolute and relative frequencies, and compared using the Chi-square test or Fisher’s exact test when appropriate. Continuous variables were presented as medians and interquartile ranges (IQRs). The Kolmogorov–Smirnov test was used to assess normality, and either parametric (independent samples *t*-test) or non-parametric (Mann–Whitney U test) methods were applied accordingly.

A binary logistic regression model was performed to identify independent predictors of ICU mortality. This model was constructed using the Enter method. Variables including age, gender, SOFA, APACHE II, presence of SIRS, presence of tracheostomy, CCI, corticosteroid administration, Gram-stain, presence of MDR/XDR/PDR, ARDS, AKI, and septic shock were selected a priori, based on clinical relevance and the previous literature. Only those variables that demonstrated statistical significance were retained in the final model. Additionally, a Cox proportional hazard regression model was used for time-to-event survival analysis. Survival curves were constructed using the Kaplan–Meier method and compared with the log-rank test. ROC curve analysis was used to determine the predictive value of selected clinical parameters. A two-sided *p*-value < 0.05 was considered statistically significant.

## 3. Results

A total of 156 ICU patients were included in our study as shown in Table 1. Patients in the COVID-ICU group were significantly older than those in the non-COVID-ICU group (median age 64 vs. 50 years, *p* < 0.0001), while COVID-ICU patients presented with more severe disease as reflected in the significantly higher severity scores (SOFA and APACHE II). As expected, the primary diagnosis at admission differed substantially: respiratory tract infections were present in all COVID-ICU patients, whereas non-COVID patients were more frequently admitted for trauma, neurosurgical, or surgical conditions. Among comorbidities, arterial hypertension was more prevalent in the COVID-ICU group (*p* < 0.001), while other conditions such as diabetes and obesity showed no statistically significant differences. PLT and Hb values appeared significantly lower in non-COVID-ICU patients.

As presented in Table 2, COVID-ICU patients had significantly higher PITT bacteremia scores (*p* = 0.001), indicating more severe infections. However, the time from ICU or hospital admission to positive culture was similar between groups. The proportion of catheter site placements did not differ significantly between groups.

There were no statistically significant differences in microbiological characteristics between the groups (Table 3). Gram-negative organisms predominated in both groups, particularly *A. baumannii* and *K. pneumoniae*. Fungal and bacterial resistance patterns were also comparable across groups. Pathogens related to second and third episodes of bacteremia are depicted in Supplementary Table S1.

As shown in Table 4, ICU length of stay was slightly shorter in the COVID-ICU group (median: 30 vs. 34.5 days, *p* = 0.013), although the interquartile ranges suggest substantial variability, particularly among non-COVID patients (IQR: 60 days), possibly due to causes of admission in this group.

**Figure 1:**
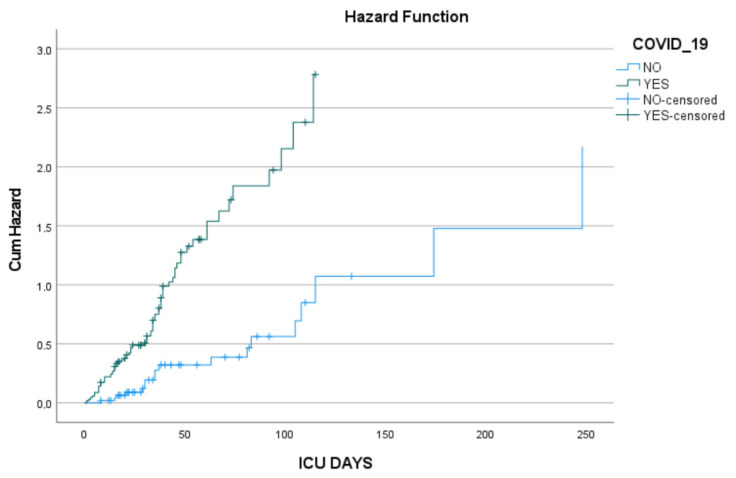
Cumulative hazard curves for ICU patients stratified by COVID-19 status. Patients with COVID-19 (green line) showed a consistently higher cumulative hazard of death compared to those without COVID-19 (blue line). Censored observations are indicated by crosses.

Kaplan–Meier survival analysis revealed a statistically significant difference in ICU survival between patients with and without COVID-19 (Log-rank *p* < 0.001). The median ICU survival time was 34 days (95% CI: 28.9–39.1) in the COVID-positive group and 105 days (95% CI: 67.2–142.8) in the non-COVID group. These findings indicate that COVID-19 was associated with markedly reduced ICU survival and suggest a need for early and intensive management in this subgroup (Figure 1).

Further survival analysis based on SOFA score categories demonstrated strong prognostic stratification (Log-rank χ^2^ = 54.741, *df* = 11, *p* < 0.001). Patients with lower SOFA scores (e.g., SOFA 4: mean survival = 195.5 days) had substantially longer ICU survival than those with higher scores (e.g., SOFA 13: median survival = 8 days), highlighting SOFA score as a strong predictor of ICU outcome.

Kaplan–Meier analysis stratified by SOFA score showed substantial variation in ICU survival. For several categories with lower SOFA scores (e.g., SOFA 4 or 5), the median survival time was not reached, as the cumulative survival remained above 50% throughout the follow-up. In these cases, mean survival was reported instead, with notably higher values (e.g., 195.5 days for SOFA 4). This suggests that lower SOFA scores are associated with significantly longer ICU survival (Figure 1).

Survival did not significantly differ by initial isolated pathogen (Log-rank χ^2^ = 3.753, *df* = 4, *p* = 0.441), although some numerical trends were observed. Notably, *E. faecium* was associated with the lowest median survival (34 days), while the “Other” category showed the highest estimated mean survival (140.6 days), albeit with wide variability.

**Figure 2:**
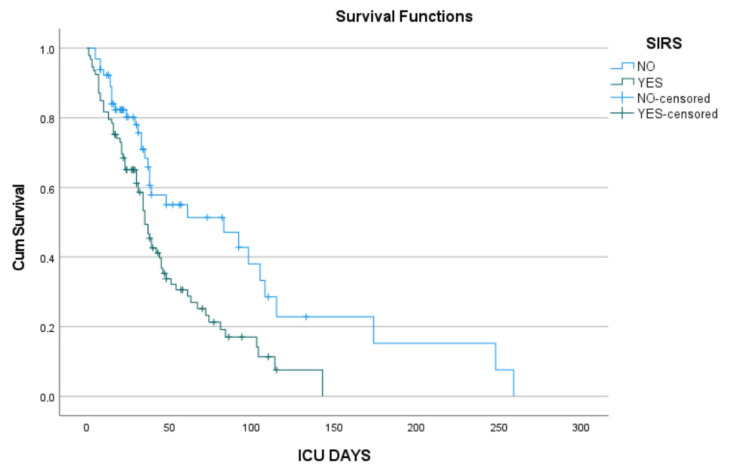
Kaplan–Meier survival curves showing the cumulative survival probability of ICU patients over time stratified by the presence of systemic inflammatory response syndrome (SIRS). Patients without SIRS (blue line) demonstrated higher survival rates compared to those with SIRS (green line). Censored observations (patients whose time of death is not known) are marked with crosses. The survival probability decreased more rapidly in patients with SIRS, indicating worse outcomes compared to those without SIRS.

No significant survival difference was found between male and female patients (Log-rank *p* = 0.429), nor between patients who did or did not receive corticosteroids (Log-rank *p* = 0.159), suggesting that neither gender nor corticosteroid use was independently associated with ICU survival. However, the presence of SIRS at admission was significantly associated with reduced survival (Log-rank χ^2^ = 9.141, *p* = 0.002). Patients without SIRS had notably longer ICU stays (mean survival = 94.6 days; median = 83 days) compared to those with SIRS (mean = 48.4 days; median = 35 days), underscoring SIRS as a potential negative prognostic factor (Figure 2).

### 3.1. Logistic Regression Analysis

A binary logistic regression analysis was performed to identify independent predictors of ICU mortality. The final model included age and SOFA score. The model demonstrated good calibration (Hosmer–Lemeshow *p* = 0.360) and explained Nagelkerke R^2^ = 0.431) with an overall classification accuracy of 80.4%. This indicates that the model had excellent ability to correctly identify patients who died (high sensitivity) and good performance in recognizing survivors (moderate specificity), which is clinically valuable in predicting ICU outcomes. Moreover, the Hosmer–Lemeshow test gives *p* = 0.360, indicating good model fit (non-significant result). Two variables emerged as significant predictors of ICU mortality:

- Age (B = 0.071, *p* < 0.001; OR = 1.074; 95% CI: 1.042–1.107;): Each additional year of age was associated with an 7.4% increase in the odds of death, holding other variables constant.
- SOFA score (B = 0.393, *p* < 0.001; OR = 1.481; 95% CI: 1.190–1.843;): Each one-unit increase in SOFA was associated with a 48.1% increase in the odds of death.

### 3.2. Cox Proportional Hazards Analysis

A multivariate Cox regression model including COVID-19 status and SOFA score was performed to assess independent predictors of ICU survival. The presence of COVID-19 was associated with a significantly increased hazard of death (HR = 3.043, 95% CI: 1.745–5.305, *p*< 0.001). Similarly, higher SOFA scores were significantly associated with increased mortality risk (HR = 1.142, 95% CI: 1.053–1.239, *p* = 0.001).

Further survival analysis based on SOFA score categories (low: 0–5, moderate: 6–9, high: ≥10) demonstrated a statistically significant difference in ICU survival (Log-rank χ^2^ = 8.464, df = 2, *p* = 0.015). Patients with lower SOFA scores had the longest mean ICU survival (61.9 days), compared to moderate (35.2 days) and high (45.4 days) groups. Median survival followed a similar pattern, confirming the prognostic utility of SOFA stratification.

These findings indicate that both COVID-19 infection and severe organ dysfunction at ICU admission are strong independent predictors of worse survival outcomes.

## 4. Discussion

We performed a retrospective analysis to assess the epidemiology, antimicrobial resistance profiles, clinical outcomes, and potential differential risk factors for MDR BSIs in patients admitted to COVID versus non-COVID ICUs. The study covered the period from 2020 to 2022, encompassing most of the COVID-19 pandemic, prior to the World Health Organization’s declaration of the end of the global Public Health Emergency of International Concern (May 2023). We found that COVID-ICU patients were older, had more comorbidities, and were in more severe condition than non-COVID-ICU patients. However, incidence of pathogens and timing and management of MDR-related bacteremia did not seem to differ between COVID and non-COVID ICU. In this setting, mortality was significantly higher in COVID ICU patients, while severity and presence of SIRS was independently associated with reduced survival. Although MDR infection did not independently predict mortality in the multivariate Cox model, we observed that COVID-19 patients with MDR BSI had prolonged ICU stays and higher organ dysfunction scores. This suggests that MDR infection contributes to poorer clinical trajectories even when not an independent predictor of mortality.

COVID ICU patients were significantly older and had higher CCI than non-COVID ICU population. There is a complex interplay between age, comorbidities, and COVID-19 outcomes. While age is a significant factor, comorbidities play a crucial role in determining mortality risk [15]. Hypertension, diabetes, and cardiac conditions are among the most common comorbidities observed in COVID-19 patients [16,17]. Interestingly, cardiac conditions showed a protective effect against mortality in younger patients without pulmonary comorbidities [18]. However, pulmonary conditions significantly increased mortality risk, especially when co-occurring with cardiac conditions [18]. The presence of multiple comorbidities was associated with increased disease severity and mortality [16]. Older patients with comorbidities had higher ICU admission rates and mortality [17].

No significant sex differences were noted between the two ICUs, although previous research indicates significant sex differences in COVID-19 outcomes (including ICU), with men experiencing higher severity and mortality rates compared to women [19,20,21]. Sex hormones play a crucial role in immune responses and disease progression, with estrogen potentially offering protective effects in women [22]. As expected, the majority of patients in COVID-ICU were admitted due to infection, i.e., COVID-19, while trauma and surgical pathology were leading causes of admission in non-COVID-ICU. This is in line with previous data in the literature, although causes exhibit significant variations across demographics and hospital types [23,24]

COVID-ICU patients presented more severe disease as this reflected in higher SOFA, APACHE, and PITT bacteremia scores, but also in higher CRP values. At the moment, no comparison is available in terms of severity scores between COVID and non-COVID patients. However, several studies have evaluated the performance of conventional severity scores in predicting outcomes for COVID-19 ICU patients. The APACHE II, APACHE IV, SAPS II, SOFA, and qSOFA scores demonstrated good discriminative power for ICU mortality, although these scores may underestimate mortality in COVID-19 patients [25]. The APACHE II score showed the best performance in predicting ICU admission, with an AUC of 0.851 [25]. A novel COVID-19 severity score (CSS) based on inflammatory markers outperformed APACHE IV in predicting mortality [26]. Additionally, the 4C Mortality Score and VACO index were significantly higher in non-survivors [27]. These findings suggest that while conventional severity scores are useful for COVID-19 patients, new or adapted scoring systems may be needed to improve mortality prediction in this population.

Multi-organ failure, as a result of systemic inflammatory response, is common in ICU patients [28]. The pathophysiology involves a complex inflammatory response, including microcirculatory alterations, immunological changes, and endothelial dysfunction, leading to cellular hypoxia and apoptosis [29]. In the context of SARS-CoV-2, multiorgan dysfunction may result from direct viral pathogenesis and indirect mechanisms such as cytokine storm, endothelial dysfunction, and coagulation abnormalities [30]. The involvement of multiple organs complicates patient care, prolongs hospitalization, and increases mortality rates [30]. Of note, in our cohort, COVID-ICU patients presented significantly more acute coronary syndrome events. SARS-CoV-2 infection can lead to various cardiovascular complications, including acute myocardial injury, myocarditis, heart failure, arrhythmia, and thromboembolic events [31,32]. The virus enters the cardiovascular system by binding to ACE2 receptors, causing systemic inflammation and direct myocardial injury [31]. Pre-existing cardiovascular disease is associated with increased COVID-19 severity and mortality. Even after recovery, some patients may experience post-acute COVID syndrome with cardiopulmonary manifestations [32]

Central line-associated bloodstream infections (CLABSIs) remain a significant concern in ICUs, with reported rates ranging from 1.12 to 17.04 per 1000 catheter-days [33]. Risk factors for CLABSI include immunosuppression, prolonged catheter duration, and use of double-lumen catheters [33]. Studies have also shown that site of insertion is associated with diverse rates of CLABSIs and catheter colonization [34]. Femoral is the site most commonly infected, while the subclavian site generally demonstrates the lowest colonization rates [35]. No difference in CVC insertion sites was noted between groups, with the majority placed in the inner jugular vein.

At the time of positive blood cultures, COVID-ICU patients were in more severe condition than the non-COVID ICU population. However, timing of bacteremia presentation since admission to hospital or ICU did not differ between groups, while both were promptly managed with appropriate antibiotic therapy. Timely initiation of adequate antimicrobial therapy is pivotal in order to reduce mortality [14,36].

BSIs in COVID-19 patients admitted to ICUs show distinct epidemiological patterns compared to non-COVID-19 patients. COVID-19 patients experience higher rates of respiratory and primary BSIs, with increased prevalence of *Enterococcus* and *Acinetobacter* species [12]. Risk factors for BSIs in COVID-19 patients include prior hospitalization, multiple comorbidities, central venous catheters, severe SARS-CoV-2 pneumonia, and lack of vaccination [37]. In our report, no difference was noted between identified pathogens in COVID and non-COVID ICU patients, although predominance of *Acinetobacter*, *Klebsiella*, and *Enterococci* was noted. BSIs significantly worsen outcomes for COVID-19 patients, with increased mortality rates observed in both ICU and non-ICU settings [12,37].

Fungal co-infections emerged as a significant and life-threatening complication during the COVID-19 pandemic, with critically ill patients experiencing substantially increased risk of opportunistic fungal diseases [38,39,40]. Invasive fungal infections were common in patients with prolonged hospital stays, particularly in later BSI episodes, likely due to extended antibiotic use, immunosuppression (including corticosteroid administration and/or tocilizumab), and invasive procedures. A meta-analysis estimated a 5% prevalence among ICU patients, with aspergillosis and candidiasis predominating [41,42]. Major risk factors include older age, chronic diseases, immunosuppressive therapy, and prolonged ICU interventions [41,42].

The pandemic has led to a rise in MDR organisms, particularly in settings where antimicrobial resistance was already established [43]. MDR bacteremias are associated with significantly poorer outcomes compared to susceptible infections, including mortality and ICU stays. The presence of MDR pathogens often necessitates the use of last-resort antimicrobials such as colistin or tigecycline, which are less effective and more toxic [44].

Studies suggest that COVID-19 patients in ICUs may have a higher risk of MDR bacterial infections compared to non-COVID-19 patients. Factors contributing to this increased risk include prolonged ICU stays, invasive procedures, and immunomodulatory treatments [45]. *A. baumannii* and *K. pneumoniae* were commonly identified MDR pathogens in COVID-19 patients [46,47]. Several studies have shown that COVID-19 patients with MDR BSIs had higher mortality rates, longer ICU stays, and increased likelihood of septic shock [7,12]. However, the impact of MDR infections on patient outcomes varied across studies. While one study found that MDR infections correlated with poor clinical outcomes, another reported lower ICU mortality among MDR-infected patients [48]. The conflicting results highlight the need for further research to better understand the relationship between MDR infections and COVID-19 patient outcomes, as well as to develop targeted preventive measures and personalized treatments [45].

Comparative studies reveal that the COVID-19 pandemic was associated with increased MDR and PDR pathogen incidence, particularly in ICU settings. Post-pandemic data from Romania and China demonstrate rising resistance in *Klebsiella*, *Acinetobacter*, and *E. coli* isolates, especially to last-line antibiotics, while some carbapenem-resistant strains declined, potentially due to strengthened infection control measures [49,50]. A global meta-analysis suggests stable overall MDR prevalence, except for increases in low-income regions [45,51]. Evidence also indicates that COVID-19 patients, though not more frequently colonized, face a higher risk of ICU-acquired MDR infections, emphasizing the need for targeted post-pandemic surveillance and additional meta-analyses in ICU contexts [45,51].

Mortality was found to be higher in COVID-ICU vs. non-COVID ICU patients; however, the authors of the present report acknowledge that mortality remains a complex outcome that cannot be solely attributed to one parameter only, including MDR BSI. The elevated mortality in the COVID-ICU cohort may be attributed to the severity of respiratory failure, higher incidence of multiorgan dysfunction, and longer ICU stays predisposing the patient to secondary infections. According to a 2023 study, increased mortality in COVID ICU is associated with factors such as the lack of effective antiviral treatment during the first waves, various comorbidities and old age, delay in the use of remdesivir or tocilizumab when indicated, fungal co-infections, multiorgan dysfunction, and ventilator-associated complications [52].

Nonetheless, in this context, the impact of the COVID-19 pandemic on ICU mortality for non-COVID patients has been found to vary across studies and settings. One study found no significant difference in mortality between ventilated COVID-19 and non-COVID-19 patients, (43.8% vs. 40%, *p* > 0.05), although COVID-19 patients had longer ICU stays and ventilation periods [53]. Similarly, Bologheanu et al. [54] reported no significant difference in ICU mortality between non-COVID-19 patients during the pandemic and pre-pandemic controls (8.5% vs. 5.2%, *p* = 0.248). However, other research indicated increased mortality for non-COVID ICU patients during the pandemic. A population-based study in Ontario reported a 7.9% relative increase in all-cause in-hospital mortality for non-COVID ICU patients during the pandemic [55]. An international study observed increased ICU mortality for non-COVID patients in middle-income countries but decreased mortality in high-income countries [56]. These studies suggest that while COVID-19 ICU patients may require longer stays and more intensive care, their mortality rates are not consistently higher than those of non-COVID-19 ICU patients or pre-pandemic ICU patients. Factors such as ICU surge conditions may have contributed to increased mortality odds [57].

This study bears inherent limitations due to its retrospective nature. No information on antibiotic or antiviral scheme (regimen, dosage, and duration) was recorded. Also, no data on potential COVID-19 immunization status, prior infection, or variant was available that could have affected the outcome or presence of systemic inflammatory response [58]. Although this study primarily first assessed the MDR BSI episode recorded in an ICU setting, and no differences were noted in pathogen epidemiology between COVID and non-COVID patients, as well as, incidents of second and third bacteremia episodes, one cannot overlook the potential impact of combination of sequential pathogen BSIs, especially in the context of long ICU hospitalizations. Another key limitation of the study is the arithmetic imbalance between COVID-19 and non-COVID-19 patient groups, as the number of non-COVID-19 patients was approximately half that of COVID-19 patients. This difference may have influenced the statistical power and interpretation of between-group comparisons. To minimize the impact of unequal group sizes, we performed weighted statistical analyses, to ensure that each group contributes proportionally to the overall analysis, thereby reducing bias introduced by arithmetic imbalance. Additionally, our single-center design contributed to the small size of the sample of patients and also limits the generalizability of findings.

The pandemic’s impact on non-COVID ICU patients appears to be influenced by various factors, including healthcare resources, policy responses, and ICU strain, highlighting the need for comprehensive pandemic planning to maintain quality care for all critically ill patients. In this context, understanding differences in MDR BSIs between the COVID and non-COVID critically ill population is essential for optimizing infection control strategies, guiding empirical antibiotic therapy, and improving patient outcomes in both pandemic and post-pandemic critical care settings.

## Figures and Tables

**Table 1:** Population characteristics.

	COVID ICU (n = 106)	Non-COVID ICU (n = 50)	Statistical Significance
Epidemiology			
Age (years) (median, IQR)	64 (15)	50 (33)	<0.0001
Sex: male (n, %)	73 (68.9)	40 (80)	0.146
Cause of ICU admission			
Infection n (%)	105 (99.1)	8 (16)	<0.001
Trauma n (%)	1 (0.9)	10 (20)	<0.001
Neurosurgical Pathology n (%)	0 (0)	16 (32)	<0.001
Surgical Pathology n (%)	0 (0)	11 (22)	<0.001
Other n (%)	0 (0)	5 (10)	<0.001
Comorbidities			
CCI Score (median, IQR)	2 (2)	1 (4)	0.016
Diabetes Mellitus n (%)	29 (27.36)	7 (14)	0.100
Heart Failure n (%)	1 (0.9)	1 (2)	0.540
Chronic Kidney Disease n (%)	1 (0.9)	1 (2)	0.540
Coronary Heart Disease n (%)	5 (4.8)	6 (12)	0.177
Dyslipidemia n (%)	34 (32)	9 (18)	0.100
Thyroid Dysfunction n (%)	12 (11)	1 (20)	0.063
Arterial Hypertension n (%)	54 (51)	10 (20)	<0.001
Cerebrovascular Disease n (%)	2 (1.8)	2 (4)	0.594
Atrial Fibrillation n (%)	6 (5.6)	1 (2)	0.431
Chronic Obstructive Pulmonary Disease n (%)	12 (11)	3 (6)	0.390
Dementia n (%)	2 (1.8)	0 (0)	1.000
Solid Organ Malignancy n (%)	9 (8.5)	9 (18)	0.107
Hematological Disease n (%)	2 (1.9)	1 (2)	1.000
Chronic Liver Disease n (%)	4 (3.8)	1 (2)	1.000
Obesity n (%)	42 (50.6)	16 (41)	0.339
Neuropsychiatric Disease/Drug Overuse n (%)	8 (7.5)	6 (12)	0.379
Autoimmune n (%)	3 (2.8)	1 (2)	0.665
Severity Indices			
SOFA (median, IQR)	8 (2)	7 (4)	<0.001
APACHE II (median, IQR)	20 (4)	19.50 (5)	0.012
Presence of SIRS n (%)	67 (63.2)	24 (48)	0.083
Laboratory Values			
WBC K/μL (median, IQR)	15,560 (9865)	12,565 (10,508)	0.170
PLT K/μL (median, IQR)	199,500 (194,000)	177,000 (202,500)	0.001
Hb mg/dL (median, IQR)	11.1 (4.1)	9 (4.3)	0.005
CRP mg/dL (median, IQR)	7.02 (13.2)	2.83 (13.32)	0.033
Ferritin ng/mL (median, IQR)	1306 (1750)	992.5 (846)	0.310
Presence of Organ Failure upon ICU admission			
Acute Respiratory Distress Syndrome n (%)	2 (1.9)	3 (6.4)	0.175
Acute Renal Injury n (%)	41 (39.4)	11 (23.9)	0.093
Disseminated Intravascular Coagulation n (%)	1 (1)	1 (2.1)	0.527
Septic Shock n (%)	29 (27.9)	11 (22.9)	0.559
Acute Coronary Syndrome n (%)	33 (31.7)	5 (10.6)	0.008

ICU: intensive care unit; IQR: interquartile range; CCI: Charlson Comorbidity Index; SOFA: Sequential Organ Failure Assessment; APACHE II: Acute Physiology and Chronic Health Evaluation II; SIRS: systemic inflammatory response syndrome; WBC: white blood cells; PLT: platelets; Hb: hemoglobin; CRP: C-reactive protein; Other: stroke, obstetric complication.

**Table 2:** Bacteremia-related characteristics.

	COVID ICU (n = 106)	Non-COVID ICU (n = 50)	Statistical Significance
PITT (median, IQR)	8 (0)	6 (2)	0.001
Days from ICU admission to (+) culture (median, IQR)	7 (9)	12 (18)	0.060
Days from hospital admission to (+) culture (median, IQR)	15 (18)	15.5 (17)	0.524
Days from (+) culture to initiation of appropriate antibiotic therapy (median, IQR)	0 (2)	0 (3)	0.129
CVC position			0.988
Jugular n (%)	95 (89.6)	45 (90)	
Femoral n (%)	7 (6.6)	3 (6)	
Subclavian n (%)	4 (3.8)	2 (4)	

ICU: Intensive Care Unit; IQR: Interquartile Range; PITT: Pitt Bacteremia Score; CVC: Central Venous Catheter.

**Table 3:** Pathogen-related Characteristics.

	COVID ICU (n = 106)	Non-COVID ICU (n = 50)	Statistical Significance
Pathogen Identified			0.441
Gram (+) n (%)	6 (5.7)	4 (8)	
Gram (−) n (%)	96 (90.6)	42 (84)	
Fungi n (%)	4 (3.8)	4 (8)	
Monomicrobial n (%)	106 (100)	50 (100)	
Type of pathogen n (%)			0.198
*K. pneumoniae*	36 (34)	19 (38)	
*A. baumannii*	59 (55.7)	23 (46)	
*E. faecium*	6 (5.7)	2 (4)	
*Candida* sp.	4 (3.8)	3 (6)	
Other	1 (1.9)	3 (6)	
Bacterial Resistance Mechanisms			0.155
CRAB n (%)	59 (59)	23 (52.3)	
CRE n (%)	34 (34)	20 (45.5)	
MR-CNS n (%)	0 (0)	0 (0)	
VRE n (%)	6 (6)	0 (0)	
Fungal resistance phenotype			1.000
Azole resistance n (%)	3 (75)	3 (75)	
Echinocandin resistance n (%)	1 (25)	1 (25)	
Amphotericin B resistance n (%)	0 (0)	0 (0)	

ICU: intensive care unit; CRAB: carbapenem-resistant *Acinetobacter baumannii*; CRE: carbapenem-resistant *Enterobacteriaceae*; MR-CNS: methicillin-resistant coagulase-negative *Staphylococci*; VRE: vancomycin-resistant *Enterococcus*; Other: *S.lugdunensis*, *A. lwoffii.*

**Table 4:** Bacteremia-related outcomes.

	COVID ICU (n = 106)	Non-COVID ICU (n = 50)	Statistical Significance
Days from initiation of appropriate antibiotic therapy to (−) culture (median, IQR)	1 (14)	2 (13)	0.587
Length of stay (median, IQR)	30 (30)	34.5 (60)	0.013
Mortality (Death) n (%)	79 (74.5)	19 (38)	<0.001

IQR: interquartile range; COVID: coronavirus disease; ICU: intensive care unit.

## Data Availability

The data generated and analyzed during the current study were retrieved from the hospitals’ archives and the electronic patient record system for laboratory results. Due to patient privacy and ethical considerations, these data are not publicly available. Access may be granted upon request to the corresponding author, which is subject to approval by the relevant institutional review boards.

## Supplementary Materials

The following supporting information can be downloaded at https://www.mdpi.com/article/10.3390/pathogens14111152/s1, Table S1: 2nd and 3rd bacteremia related pathogens.

## Abbreviations

The following abbreviations are used in this manuscript:

Abbreviation	Full Term
AKI	Acute Kidney Injury
AMR	Antimicrobial Resistance
ARDS	Acute Respiratory Distress Syndrome
APACHE II	Acute Physiology and Chronic Health Evaluation II
BLSI	Bloodstream Infection (used interchangeably with BSI)
BSI	Bloodstream Infection
CCI	Charlson Comorbidity Index
CLABSI	Central Line-Associated Bloodstream Infection
COVID-ICU	COVID-19 Intensive Care Unit
CRE	Carbapenem-Resistant *Enterobacteriaceae*
CRAB	Carbapenem-Resistant *Acinetobacter baumannii*
CRP	C-Reactive Protein
CNS	Coagulase-Negative Staphylococci
COVID-19	Coronavirus Disease 2019
CVC	Central Venous Catheter
CDC	Centers for Disease Control and Prevention
ECDC	European Center for Disease Prevention and Control
EUCAST	European Committee on Antimicrobial Susceptibility Testing
GDPR	General Data Protection Regulation
Hb	Hemoglobin
HR	Hazard Ratio
ICU	Intensive Care Unit
IQR	Interquartile Range
IV	Intravenous
MDR	Multidrug-Resistant
MIC	Minimum Inhibitory Concentration
MR-CNS	Methicillin-Resistant Coagulase-Negative Staphylococci
OR	Odds Ratio
PDR	Pandrug-Resistant
PLT	Platelets
PITT	Pitt Bacteremia Score
ROC	Receiver Operating Characteristic
SAPS II	Simplified Acute Physiology Score II
SIRS	Systemic Inflammatory Response Syndrome
SOFA	Sequential Organ Failure Assessment
VRE	Vancomycin-Resistant *Enterococcus*
WBC	White Blood Cells
XDR	Extensively Drug-Resistant
