# Supplementary material for: MDR Bacteremia in the Critically Ill During COVID-19: The MARTINI Study
Source: Pathogens. 2025 Nov 12;14(11):1152. doi: 10.3390/pathogens14111152 (PMC12655241; doi:10.3390/pathogens14111152)
Supplement: Supplementary file 1 [file pathogens-14-01152-s001.zip › pathogens-3917727-supplementary.pdf]

Supplementary Materials

Table S1. 2nd and 3rd bacteremia related pathogens.

	**COVID-ICU** ***n*** **= 106**	**Non COVID-ICU** ***n*** **= 50**	**Statistical Significance**
2nd bacteremia *n* (%)			0.236
K. pneumoniae	13(12.3)	6(12)	
A. baumannii	11(10.4)	2(4)	
E. faecium	8(7.5)	6(12)	
Candida sp.	9(8.5)	10(20)	
Other	17(16)	8(16)	
3rd bacteremia *n* (%)			0.406
K. pneumoniae	3(2.8)	4(8)	
A. baumannii	1(0.9)	0(0)	
E. faecium	2(1.9)	3(6)	
Candida sp.	5(4.7)	1(2)	
Other	5(4.7)	2(4)	

COVID: Coronavirus Disease; ICU :Intensive Care Unit.
